# Advancements and limitations in traditional anti-cancer therapies: a comprehensive review of surgery, chemotherapy, radiation therapy, and hormonal therapy

**DOI:** 10.1007/s12672-025-02198-8

**Published:** 2025-04-24

**Authors:** Aasma Zafar, Summaiya Khatoon, Muhammad Jawad Khan, Junaid Abu, Aisha Naeem

**Affiliations:** 1 Department of Biosciences, COMSATS University, Islamabad, 45550 Pakistan; 2 College of Health Sciences, QU Health Sector, Qatar University, P.O. Box 2713, Doha, Qatar; 3 Aisha bint Hamad al-Attiyah hospital, Hamad Medical Corporation, P.O. Box 3050, Doha, Qatar; 4 Research and Graduate Studies, QU Health Sector, Qatar University, P.O. Box 2713, Doha, Qatar

**Keywords:** Cancer, Anticancer therapies, Surgery, Hormonal therapy, Antimetabolites

## Abstract

Cancer remains a major global health challenge, consistently ranking as the second leading cause of mortality worldwide. Despite significant advancements in research and technology, the need to deepen our understanding of tumor biology and improve therapeutic strategies persists. This review focuses on the progress and challenges of four traditional cancer treatment modalities: surgery, chemotherapy, radiation therapy, and hormonal therapy. Surgery, the primary method for tumor removal, has evolved with the integration of fluorescence-based technology and robotic systems, enhancing precision and minimizing collateral damage. Radiation therapy has progressed with improved focus, intensity control, and 3D technology, refining both diagnosis and treatment. Chemotherapy has advanced from natural extracts to synthesized derivatives with amplified cytotoxicity against cancer cells. Hormonal therapy has emerged as a crucial strategy for hormone-dependent cancers, restraining growth or inducing regression. Despite these advancements, each approach faces ongoing challenges. Surgery struggles with complete tumor removal due to heterogeneity. Chemotherapy contends with drug resistance and side effects. Radiation therapy grapples with precision issues and limited access in some regions. Hormonal therapy faces resistance development and quality of life impacts. This study provides a comprehensive analysis of the evolution of these traditional anti-cancer therapies, offering insights into their progress and highlighting areas for future research. By examining these modalities, we aim to underscore their relevance in the current oncology landscape and identify opportunities for improvement in cancer treatment strategies.

## Highlights

- Significant progress has been made in traditional cancer treatments, including surgery, radiotherapy, chemotherapy, and hormonal therapy, enhancing their effectiveness and precision.
- Modern techniques such as fluorescence-guided surgery, laparoscopic procedures, and robot-assisted surgery have improved tumor removal precision but may still contribute to minimal residual disease (MRD) and potential recurrence.
- Techniques like SBRT and IMRT have advanced radiation therapy by increasing targeting precision, though challenges such as resistance and immune suppression remain.
- Chemotherapeutic agents, including alkylating compounds, antimetabolites, and topoisomerase inhibitors, are essential for treatment but face issues like toxicity, resistance, and limited efficacy.
- Hormonal therapies, such as tamoxifen and ADT, are crucial for hormone-dependent cancers, but resistance mechanisms necessitate new approaches.
- Future research should focus on understanding molecular mechanisms to increase specificity, radiosensitivity and reduce cytotoxicity to drive innovations in cancer therapy.

## Introduction

Cancer, characterized by the unwanted proliferation of cells arising from an aggregation of various genetic anomalies, including dysregulation of genes and their regulators, has a deep-rooted history dating back approximately 1.98 million years. Evidence of tumors, specifically osteosarcoma, has been discovered in human fossils from South Africa, showcasing the ancient existence of this disease [1]. Historical records indicate cancer as the reported cause of death in Egyptian mummies dating back to around 1500 BC [1]. The term “cancer,” coined by the Greek physician Hippocrates, derives from the disease’s crab-like projections as it spreads into adjacent tissues [2]. In the fifteenth century, a Roman physician introduced the term “oncos” to describe swelling, highlighting early attempts to understand and categorize tumors. During that period, the only available treatment option involved herbal medication or the application of a poultice near the cancer following a restricted incision [3]. In the fifteenth century, a greater understanding of the structure and functioning of the human body, tumor biology, and the discovery of anesthesia prompted physicians to consider surgery as the most viable option for cancer treatment.

**Fig. 1:**
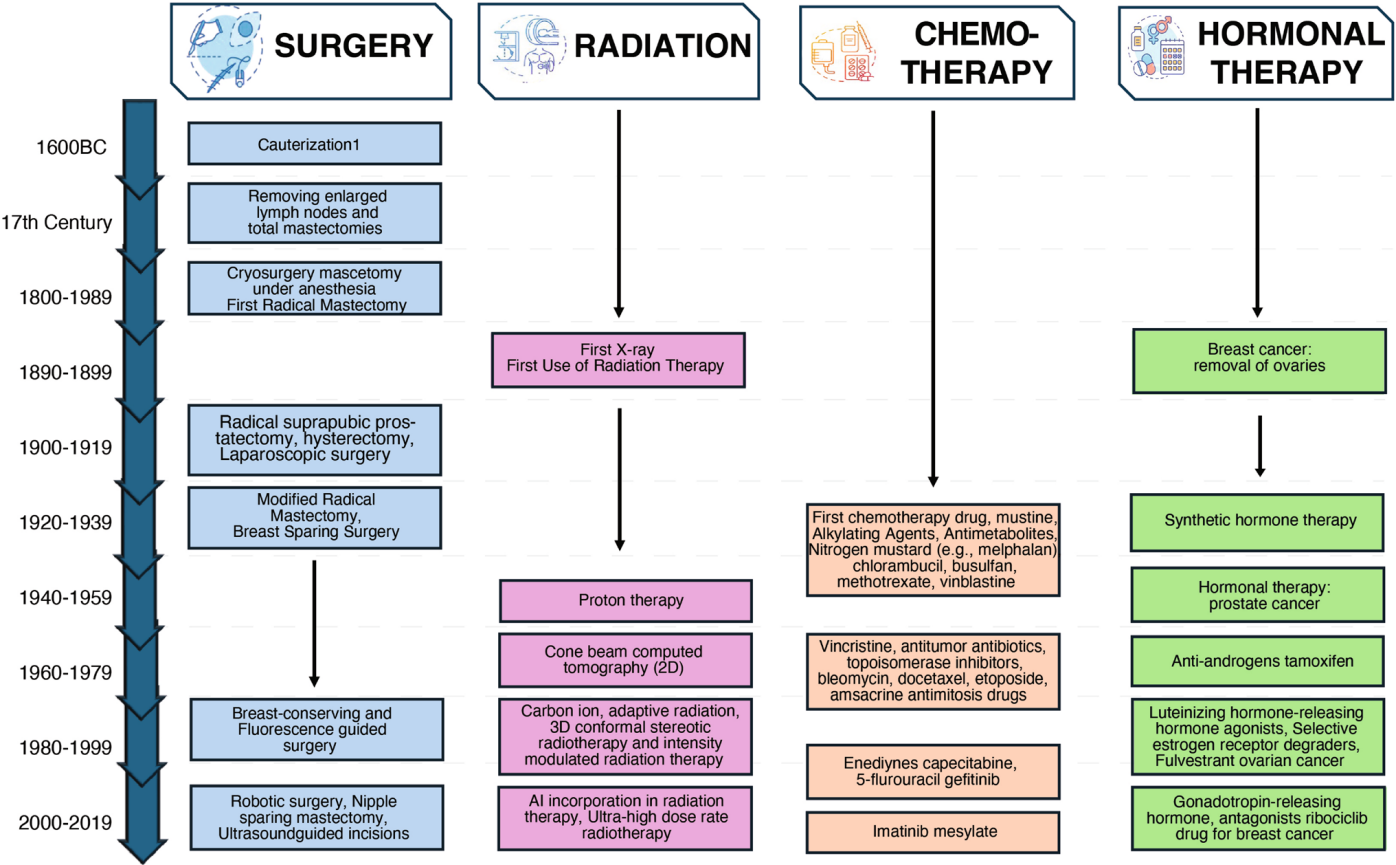
Evolution and milestones of traditional anticancer therapies across centuries: A comprehensive overview of development and progress

The persistent rise in cancer-related deaths, coupled with the inherent risks and severe side effects of existing treatments, highlights the urgent need for more effective and personalized options. Despite the emergence of advanced therapies such as immunotherapy and gene therapy, traditional treatments remain the most commonly adopted due to their cost-effectiveness and widespread use. Therefore, ongoing efforts to improve and refine conventional cancer treatments—such as surgery, radiotherapy, chemotherapy, and hormonal therapy—remain essential, as illustrated in Fig. 1. This review aims to provide a comprehensive overview of the advancements and limitations of these traditional therapies. By exploring their current state, evolutionary progress, and enduring challenges, we seek to identify areas of unmet need and highlight potential avenues for future research and innovation.

## Surgery

Surgery is a time-honored and fundamental practice for treating and diagnosing solid malignancies. In diagnostics, cancerous tissue biopsy is the optimal choice for subtyping and grading cancers; however, insufficient sample size and routine painful monitoring limit its effectiveness. Recently developed liquid biopsy offers non-invasive monitoring of cancer-specific biomarkers such as circulating tumor DNA (ctDNA), circulating tumor cells (CTC), and Extracellular vesicles (EV), which are released by primary tumors for metastasis [4]. However, identifying novel cancer-specific biomarkers and highly sensitive technology to detect the minor quantity of biomarkers is indispensable for effective diagnosis at early stages of disease.

Excision surgery is performed to achieve complete clearance of tumor cells. However, despite apparently successful resection, residual disease often remains in the clearance margins, microscopic deposits, and micro-metastases (stromal or hematogenous), indicating the presence of minimal residual disease (MRD) [5]. Studies have shown that MRD can progress rapidly, and the surgical removal of the tumor mass often correlates with this progression [6]. The aggressive nature of MRD can be attributed to various factors, including the surgical technique itself and the trauma-induced environment, which may foster conditions that promote cancer cell proliferation. Perioperative factors, such as the use of opioids for pain management and blood transfusions, may further contribute to this phenomenon [7].

The post-surgical introduction of immunosuppressors to aid wound healing facilitates the evasion of tumor cells from the immune system. This results in decreased natural killer (NK) cytotoxicity and compromised macrophage function linked to increased tumor growth post-surgery [7]. Surgical intervention may also reduce circulating dendritic cells (DC), which are crucial for immune surveillance following tumor removal [7].

Additionally, removing antiangiogenic factors released by the primary tumor facilitates neovascularization potential in micro-metastases at other sites, exacerbating disease progression. The surgical removal of the primary tumor frees MRD from inhibitory control, known as concomitant tumor resistance, leading to the unhindered growth of metastatic foci [7]. Proangiogenic factors secreted by primary tumor cells promote the establishment of new vessels at the primary site, fostering cancer progression [7]. Additionally, removing antiangiogenic factors released by the primary tumor facilitates neovascularization potential in micro-metastases at other sites, sparking disease progression. The surgical removal of the tumor reduces levels of antiangiogenic factors and increases the presence of angiogenic factors. This shift empowers previously inactive micro-metastases to undergo angiogenesis, ultimately exacerbating the progression of the disease [7].

To address the complications associated with traditional surgery, numerous technological advancements have been introduced over the years, shifting from open surgery to minimally invasive techniques, such as laparoscopic surgery, which reduces blood loss and lowers morbidity (Fig. 1). Cryo-technology and laser techniques have improved surgical precision, while fluorescence-guided surgery (FGS) allows surgeons to distinguish between tumor and normal cells, helping to delineate tumors and avoids leaving behind cancerous tissue or damaging surrounding healthy cells [8]. Video-assisted laparoscopic surgery (VALS) enhances precision by offering real-time visualization, though its two-dimensional view and limited maneuverability in tight spaces present challenges [9]. The advent of robotic surgery has provided surgeons with greater control, precision, and flexibility, while allowing them to view the surgical area in magnified three-dimensional images [10]. Over time, the autonomy of surgical robots has increased, evolving from mere assistants to systems capable of developing personalized surgical strategies [11]. This increased autonomy enables surgeons to plan and execute complex surgeries with enhanced precision and tailored approaches.

Although these technological advancements have allowed surgeons to reduce the chances of minimal residual disease (MRD) and surgical stress, advancements like robotic surgery have minimized errors in the removal of tumor tissue, which can sometimes trigger the emergence of metastatic loci. Surgical intervention also carries potential complications, including infections, impaired quality of life, and challenges in post-surgical recovery. Many recent [12] and historical studies [13] highlighted no survival advantage over anticipated clinical management by chemotherapy. Some conditions, such as heart disease, bleeding disorders, obesity, chronic obstructive pulmonary disease (COPD), asthma, other lung conditions, and kidney problems, can increase the risk of complications during surgery [5]. Hence, understanding the possible outcomes of surgery along with the body and immune response to clearance of tumor tissue is essential for making informed decisions.

## Radiotherapy

Radiotherapy is a highly effective cancer treatment that uses high-energy particles, such as X-rays, gamma rays, and protons, to destroy cancer cells by damaging their DNA. Since the discovery of X-rays, these have been utilized to treat cancers of various origins [14]. Technological advancements and an improved understanding of tumor biology have led to significant progress in radiotherapy treatment incorporating techniques such as 3D conformal stereotactic (body) radiotherapy (SBRT), intensity-modulated radiation therapy (IMRT), and enhanced imaging systems like image-guided radiation therapy (IGRT) [15] contributing to increased overall survival (OS) rates across various cancer types.

Historically, limited tolerance of healthy cells hindered widespread radiotherapy use. Still, recent scientific progress has expanded its application by maximizing cancer cell sensitivity while increasing the tolerance range of healthy cells. Recognizing radiation sensitizers and protectors has allowed for diverse applications, adjusting treatment according to patient’s needs. Amifostine (WR2721) is a sulfhydryl-phosphorylate compound, used as a radiation protector. After dephosphorylation by alkaline phosphatase, Amifostine becomes an active metabolite that scavenges radicals and protects cells from the harmful effects of radiation [16]. Conversely, 2-deoxy-D-glucose, 6-aminonicotinamide, curcumin, and parthenolide have been developed to increase ROS levels in cancerous cells and improve the efficacy of radiation [17].

Additionally, technological development replaced the “planner imaging field” with “conformal radiotherapy,” allowing the shaping of radiation beams according to the tumor. SBRT delivers conformal and hypo-fractionated doses of radioactive beams to even small-sized tumors with superior protection to surrounding tissue [18]. IMRT has an advantage in adjusting the dosage (intensity) according to the shape and size of the cancer. Dosimetric modulation of the delivered beams according to the prescription plan shows optimistic clinical results, particularly in locally advanced tumors [19]. Ongoing research focuses on developing advanced radiation delivery systems that minimize damage to surrounding healthy tissues, such as FLASH radiotherapy, which delivers ultrahigh doses in microseconds to preserve normal tissue function [20]. Brachytherapy (internal radiotherapy) is another technique to deliver high or low dosage of radiation to unresectable tumors by directly implanting the radiation source in or near the tumor site. Irradiating tumors from inside or nearer minimizes the damage to surrounding tissues [21].

Despite these achievements, some cancer cells remain impervious to radiotherapy, and the likelihood of recurrences remains high [22]. Exposure to ionizing radiation induces hydrolysis and the creation of radioactive oxidative species (ROS), specifically hydroxyl radicals (.OH), causing DNA damage [23]. Studies emphasize the crucial role of antioxidants in determining radiotherapy success, with an increase in intracellular antioxidant concentrations limiting treatment efficiency, while decreased concentrations yield desired results [17].

Ionizing radiation leads to DNA damage, triggering DNA damage response and cell death through apoptosis, necrosis, or autophagy [24]. Double-stranded DNA repair pathways comprise homologous recombination (HR) and non-homologous end joining (NHEJ) [25]. HR, a high-fidelity repair system, utilizes DNA sequences to repair double-strand breaks (DSB). NHEJ is a faster way of double DNA repair, which ligates the broken ends of DNA without a homologous template, thus causing DNA segment deletion and/or rearrangement. Following DNA damage, damage sensing and response mechanisms activate.

Furthermore, radiation exposure increases mitochondrial ROS production, enhancing cell sensitivity through the cytosolic Rac1/NADPH oxidase system [25]. The KEAP1-NRF2 (kelch-like ECH-associated protein 1-nuclear factor erythroid 2) system plays a vital role in radiotherapy success, where NRF2, usually regulated by KEAP1, induces antioxidant gene expression under radioactive stress [26]. Experimental studies indicate that the deletion of KEAP1 results in tumor aggressiveness and resistance to radiotherapy [27], with KEAP1 mutations in non-small cell lung cancer (NSCLC) was identified as associated with an increased risk of local recurrence after radiation therapy [28].

Variations in genetics and metabolism also determine the success of radiotherapy. Enhanced consumption of glucose is one of the hallmarks of tumorigenesis. GLUT1—a widely distributed glucose transporter—is overexpressed in numerous cancers. Studies suggest the key role of GLUT1 in the prognosis of radioactivity. Increased GLUT1 expression accompanied by hypoxia and dysregulation of MAPK and PI3K/AKT pathways often confers radio-resistance to tumorous cells [29]. Likewise, fumarate hydratase (FH), a critical enzyme in the tricarboxylic acid (TCA) cycle, is implicated in hereditary and sporadic cancers due to mutations. Loss of FH activity leads to fumarate accumulation and is often linked to leiomyomata and tumor development. Fumarate binds with antioxidant glutathione to produce succinated glutathione (GSF), which is an alternative substrate to glutathione reductase (GR) and downregulates NADPH levels while promoting ROS production [30]. Understanding the potential response of cancerous cells shaped by metabolism and genetics could provide insights into therapeutic interventions for treating tumors.

Cellular response to radiotherapy is greatly influenced by its oxygen content, with hypoxia enabling cells to resist DNA damage. In contrast, increased oxygen concentration in cancer cells makes them prone to DNA damage [31]. The different oxygen tensions in tumors and normal tissues may underpin the beneficial sparing effect seen in normal tissue during radiotherapy [32]. Increased hypoxia-inducible factor 1-alpha (HIF-1α) expression in numerous cancers correlates with poor survival. It facilitates cancer cell growth through various pathways, impacting proliferation, angiogenesis, metastasis, ROS homeostasis, extracellular matrix remodeling, and pH regulation. In oropharyngeal cancer, increased HIF-1α expression promoted relapse after radiotherapy while knocking down HIF1-α enhanced sensitivity towards radiation [31].

Overall, the impact of oxygen levels, genetics, and metabolites on radiotherapy response involves complex mechanisms that influence DNA damage and HIF-1α expression, thus affecting clinical outcomes. Pre-treatment imaging utilizing techniques such as computed tomography (CT), magnetic resonance imaging (MRI), or positron emission tomography (PET) provides detailed visualizations of the tumor’s size, shape, location, and surrounding healthy tissues, laying the foundation for precise radiotherapy planning. Adaptive radiotherapy protocols enhance treatment by dynamically adjusting plans during the therapy course to account for tumor shrinkage or anatomical changes, ensuring optimal targeting and minimizing harm to healthy tissues. Comprehensive patient care complements these approaches by addressing side effects, such as fatigue and skin reactions, and providing supportive therapies to improve treatment tolerance. Together, these strategies underscore the potential of personalized radiotherapy to maximize effectiveness and improve clinical outcomes in cancer care [33].

## Chemotherapy

Chemotherapy is one of the most widely used conventional approaches to cancer treatment. It targets key cell cycle phases to induce cell death. Below, we discuss common chemotherapeutic agents, their modes of action, and the associated side effects, as summarized in Table 1.

**Table 1:** List of chemotherapeutic therapy agents for cancer treatment

Name	Mode of action	Results	Agents	Risk and issues
Alkylating agents	Covalently binds to macro-molecules having nucleophilic sites	Block replication, transcription and causes cell death	Bischloroethyl, cyclophosphamide, ifosfamide, aziridines, busulphan, 2-chloroethylnitrosoureas	Affects both tumors and normal cells; side effects; instability; systemic cytotoxicity
Antimetabolites	Converted to nucleotide analogs, which are then incorporated into DNA	Inhibition of DNA synthesis and function	6-Mercaptopurine, thioguanine, 5-fluorouracil, cytarabine, gemcitabine, decitabine	Side effects
Antibiotics	Cleaves nucleic acid or covalently binds to DNA	Inhibition of replication and transcription	Bleomycin, enediynes, mitomycin, Mithramycin, chromomycin	Development of resistance
Topoisomerase inhibitors	Target topoisomerases to prevent DNA unwinding	Inhibition of topoisomerase activity	*Camptotheca cuminata*, *Berberis aristata*, anthraquinone, naphthylquinone, phenathraquinone, ruthenium II polypyridine complex, Cu-II complex, platinum complex	Low solubility, adverse drug effects, cardiotoxicity
Mitosis inhibitors	Inhibit mitosis by disrupting microtubule polymerization or disintegration	Inhibits progression from prophase, halting mitosis	G-I, MVAC, Vincristine, Docetaxel, Paclitaxel, Cabazitaxel	Development of resistance

G-I: A growth factor, Granulocyte Colony-Stimulating Factor (G-CSF), commonly used in chemotherapy regimens to stimulate the production of white blood cells, MVAC: A chemotherapy regimen that includes four drugs: methotrexate, vinblastine, doxorubicin, and cisplatin

### Alkylating agents

**Fig. 2:**
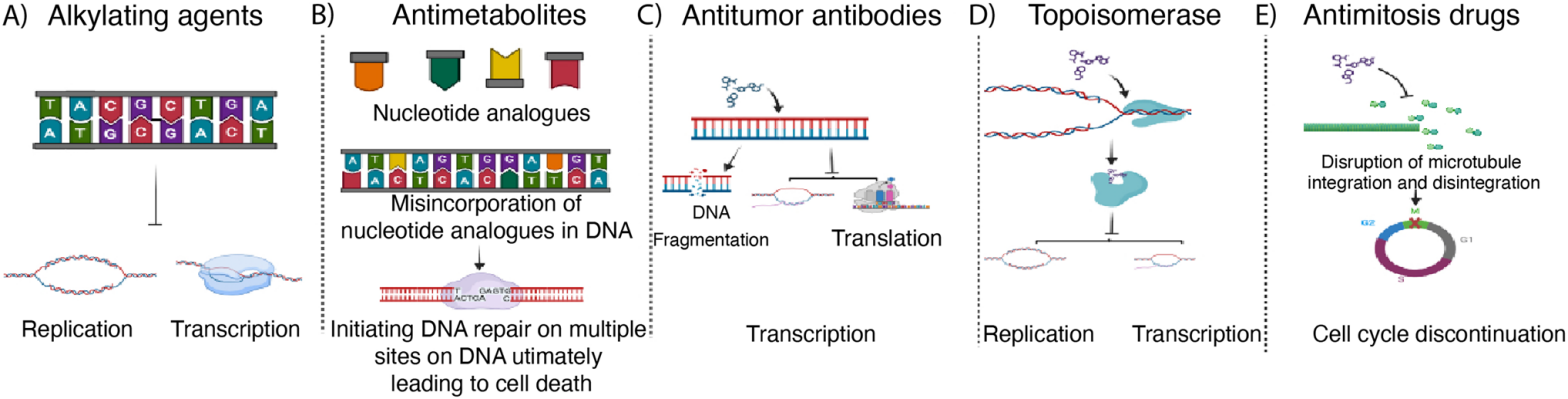
Mechanisms of action of different chemotherapeutic drugs. **A** Alkylating agents form inter-strand and/or intra-strand adducts making DNA unable to open for replication and transcription. **B** Antimetabolites utilize nucleotide analogs. Misincorporation of these nucleotide analogues initiates DNA repair on hundreds of sites simultaneously causing DNA stress and ultimate cell death. **C** Antitumor antibiotics cause DNA fragmentation and disruption of transcription and translation. **D** Topoisomerase inhibitor obstructs DNA replication, transcription, chromosomal segregation and recombination. **E** Antimitotic drugs target microtubule assembly and disassembly thereby disrupting mitosis and causing cell cycle arrest

Alkylating agents, first recognized for their clinical potential in 1942 with the introduction of nitrogen mustard gas, heralded a new era in chemotherapy. Like sulfur mustard gas used in World War I, these agents form covalent bonds with nucleophilic sites on macromolecules [34]. This interaction leads to the formation of DNA adducts, which can cause either inter-strand or intra-strand crosslinking, disrupting replication and transcription and ultimately resulting in cell death [35]. However, the inability of alkylating agents to selectively target tumor cells, as opposed to normal cells, poses profound implications for health (Fig. 2).

The nitrogen mustard family members are mechlorethamine, chlorambucil, melphalan, ifosfamide (IFO), trofosfamide and cyclophosphamide (CP). Mechlorethamine promptly activates at physiological pH, making it prone to side reactions. In contrast, CP is a relatively stable compound that requires metabolic activation through the hepatic mixed oxidase system. Its active metabolite, 4-hydroxcyclophosphamide (4-OHCP), exhibits carcinogenic, teratogenic, mutagenic, and immunosuppressive properties [35]. Ifosfamide (IFO), also requiring metabolic activation, faces slower hepatic conversion and thus necessitates a higher dosage than CP. This elevated dosage induces higher cytotoxicity, leading to the production of a toxic metabolite, acrolein, causing hemorrhagic cystitis, requiring detoxification with Mesna [36]. Aziridines, or ethylenimines, are ring-closed alkylating compounds that spontaneously activate or require enzymatic oxidative reactions. Oxidation of antineoplastic drugs is often carried out by human cytochrome P450s (CYP). Different CYP enzymes oxidize anticancerous drugs such as ifosfamide, cyclophosphamide and thiotepa to their active and cytotoxic form [37].

Busulphan (Bu), an alkyl sulphonate, directly alkylates DNA guanine, resulting in adduct formation and cytotoxicity [35]. Nitrosoureas are a group of alkylating agents that can cross the blood–brain barrier (BBB) and are cell phase non-specific and lipid soluble [38]. 2-chloroethylnitrosoureas (CENU), such as carmustine, lomustine and semustine alkylate guanine at positions N7 and O6, with cytotoxicity regulated by the DNA repair enzyme O6-alkylguanine–DNA alkyltransferase [39]. However, instability and impulsive breakdown of CENU into various products present challenges [35].

Cisplatin, a platinum-based alkylating drug, demonstrates high efficacy in treating various cancers, but its lateral effects and the development of drug resistance compromise its clinical significance [40]. Carboplatin, a cisplatin analog, emerged as a clinically active compound with reduced toxicity, overcoming renal damage and peripheral neuropathy associated with cisplatin. Nevertheless, cross-resistance with the parent molecule poses a significant limitation to its success [41]. Inside the cell, non-enzymatic conversion of cisplatin to a chemically active charged aquated species takes place, and it interacts with macromolecules like DNA. Similarly, carboplatin also produces charged aquated species but has slower rates of aquation, which lessens renal damage and neuropathy [42, 43]. Oxaliplatin, often used to treat gastrointestinal cancers, induces ribosome biogenesis stress and DNA damage response generated by interstrand cross-linkages. Moreover, Oxaliplatin is less nephrotoxic than cisplatin and carboplatin while offering comparable efficacy [44].

Temozolomide is a small lipophilic alkylating agent widely used to treat glioblastoma, a highly aggressive brain tumor. It methylates purine bases at N3 of adenine, N7 and O6 site of guanine producing N3-MeA and N7-MeG and O6-MeG, respectively [45]. N3-MeA and N7-MeG are repaired by base excision repair (BER) while O6-MeG requires DNA repair protein O6-methylguanine-DNA methyltransferase (MGMT). MGMT repairs DNA damage by removing alkyl groups from the O6 position of guanine, thereby reducing the cytotoxic effects of the drugs and contributing to treatment resistance. As such, resistance to TMZ often arises due to increased MGMT activity and alterations in drug transport mechanisms, both of which limit the drug’s efficacy [45]. Emerging clinical studies are exploring strategies to overcome resistance and address this challenge. For instance, MGMT inhibitors like O6-benzyl guanine have been investigated to sensitize tumors to the alkylating agent [46]. Additionally, alternative drug delivery approaches are being developed to enhance therapeutic outcomes and improve patient responses to treatment.

### Antimetabolites

Antimetabolites, a class of chemotherapeutic agents, work by impairing DNA replication machinery either by depletion of deoxynucleotides or incorporation of chemically modified nucleotides. Nucleotide precursor analogs are developed as anti-cancerous therapies to exploit cancer cells’ preference for the salvage pathway in purine and pyrimidine synthesis. On entering cells, these precursor nucleotide analogs transform into nucleotide analogs and prevent key enzymes from synthesizing DNA eventually prompting apoptosis [47].

Aphidicolin, an antimetabolite, engages DNA polymerase without integrating into DNA, causing a temporary delay in DNA synthesis and is not considered a good anti-cancerous candidate for long-term implications [48]. Methotrexate targets dihydrofolate reductase (DHFR), which is essential for folate biosynthesis, thus depleting folates, and reducing purine and pyrimidine synthesis. Other drugs like pemetrexed (PMX) and raltitrexed (RTX) engage in reduced folate carriers (RFC) [49]. Azacitidine is a cytidine analog that is incorporated into DNA and RNA. With preferred incorporation in RNA, azacitidine inhibits protein synthesis through the disintegration of polysomes, peaking of 80S ribosomes, and diminished ability to accept and transfer RNA. While incorporated in DNA, azacitidine blocks the cysteine methylation in newly synthesized molecules by inhibiting the activity of DNA methyltransferase (DNMT1), thus causing epigenetic changes in addition to DNA damage [50]. Clofarabine inhibits DNA polymerase and ribonucleotide reductase (RR). RR inhibition depletes the reserves of dNTP, allowing clofarabine to be incorporated into DNA. Furthermore, clofarabine disrupts the mitochondrial membrane potential and initiates apoptosis pathways [51]. Capecitabine is a fluoropyrimidine that is converted to 5-FU by thymidylate phosphorylase. 5-FU inhibits thymidylate synthase, thus causing the depletion of thymidine-5-prime monophosphate (dTMP), which is indispensable for DNA repair and replication [52]. Cladribine is an adenosine analog usually used for treating leukemia. It obstructs DNA production by inhibition of adenosine deaminase [53].

Various antimetabolites with radiosensitizing potential, including fluorouracil (FU), 5-fluoro-2′-deoxyuridine (FdUrd), and hydroxyurea (HU), have been utilized to synthesize drugs. Combining antimetabolites with radiosensitizers has shown enhanced survival outcomes for patients with solid malignancies of the gastrointestinal tract, cervix, and head and neck [54]. Thymidine analogs like 5-bromo-2′-deoxyuridine (BrdUrd) and 5-iodo-2′-deoxyuridine (IdUrd) intensify DNA damage caused by ionizing radiation, prompting cell death [55]. FU and FdUrd induce cytotoxicity and radiosensitization by inhibiting thymidylate synthase (TS), leading to the misinsertion of deoxyuridine triphosphate (dUTP) into DNA and initiating the DNA repair system. The excess of dUTP leads to multiple rounds of excision, repair and reincorporation, ultimately causing cell death [56]. FU metabolized to 5-fluorouridine monophosphate, 5-fluorouridine diphosphate, and 5-fluorouridine triphosphate disrupts RNA processing, contributing to cytotoxicity withoutradiosensitization effect [56]. The TS blockade results in an imbalance between dTTP and dATP, halting DNA synthesis and arresting cells in the early S phase, making them highly susceptible to ionizing radiation [57]. Additionally, FdUrd slows down DNA double-strand break repair, heightening cancerous cell susceptibility to radiation. Hydroxyurea (HU) inhibits ribonucleotide reductase (RR), eliminating tyrosyl free radicals crucial for reducing ribonucleotides and subsequently depleting deoxyribonucleotide triphosphates (dNTP), thus ceasing DNA synthesis. HU-induced radiosensitization proved clinically effective in cervix and head and neck cancers, improving local disease management [58].

### Antitumor antibiotics

Antitumor antibiotics are one of the earliest forms of cancer treatment, particularly those obtained from Streptomyces species. These antibiotics target multiple cell cycle phases and induce cell growth arrest (Fig. 2).

Bleomycin (BLM), a glycol peptide, cleaves nucleic acid in a sequence-selective manner through metal-dependent oxidative cleavage in the presence of oxygen. Various modifications to its functional domains have been explored to improve efficacy and reduce toxicity [59]. Studies demonstrate the inactivation of BLMs in both standard and cancerous cells by BLM hydrolase, which hydrolyzes the C-terminus amine of BLM and generates a de-amido product. However, the modification of bleomycin disaccharides to enhance tumor cell targeting aimed to define the required positioning and modification of the carbamoyl group [60]. An in-vivo study demonstrated that deglycobleomycin could be a less toxic alternative to standard bleomycin. This modification of bleomycin can potentially reduce harmful side effects, offering a promising development in cancer treatment [61].

Enediynes, natural products with antibiotic and anti-tumorous potentials, are highly cytotoxic DNA-damaging agents that have garnered attention for their potential in cancer treatment. Recent studies have focused on understanding the cytotoxic effects of enediynes and exploring strategies to enhance their efficacy [62]. They can be classified into two groups: 9-membered ring chromophore cores or 10-membered rings. The discovery of these molecules provided highly potent anti-cancer agents, as some products of 9-membered ring chromophore cores are 5–8000 times stronger than Adriamycin, a widely used anti-tumorous drug [62].

Enediynes introduce a single-strand or double-strand DNA lesion and an RNA lesion in a few cases. The binding of chromophores of enediynes with the minor groove of DNA initiates structural changes in DNA, making it susceptible to oxidation and resulting in DNA strand break. C-1027, obtained from *Streptomyces globisporus*, expressed high cytotoxicity against non-Hodgkin B cell lymphoma [63]. However, belated toxicity appeared to be a significant inadequacy for the clinical application of C-1027. Neocarzinostatin (NCS), an enediyne acquired from *Streptomyces carzinostaticus*, has shown improved uptake and reduced toxicity when conjugated with poly styrene-co-maleic acid (SMA) or its alkyl esters (SMANCS) [64].

Calicheamicin (CAL), a 10-membered enediyne derived from the bacterium *Micromonospora echinospora*, is unified with a monoclonal antibody (mAb) to enhance tumor specificity [65]. The coupling of the humanized monoclonal antibody (mAb) Gemtuzumab ozogamicin, which targets the CD33 antigen expressed in the hematopoietic system, to CAL enables precise targeting of transformed cells with minimal general cytotoxicity in acute myeloid leukemia (AML) [66].

Mitomycin (MTM), a member of the antitumor quinone class, interacts covalently with DNA to function similarly to alkylating agents [67]. The chemical composition of MTM includes aziridine, quinone, and carbamate moieties arranged in a compact pyrrolo-[1,2-*a*] indole structure which facilitates its cross-linking to DNA with greater efficacy and specificity for CpG islands. MTM-C, obtained from *Streptomyces caespitosus*, is reductively activated and converted from a non-cytotoxic prodrug to a reactive quinone methide, either enzymatically or chemically [68]. The drug is often used to treat breast, lung, colon, and head and neck cancers [69].

Differences in oxygen content and intercellular pH between normal and cancerous cells results in varied responses to MTM, leading to more significant cytotoxicity in cancerous cells [70]. Hypoxic conditions in tumors create an ideal environment for activating MTM-C, making it functional in the oxygen-deficient regions of solid tumors. FR900482, produced by *Streptomyces sandaensis* exhibits decreased hematotoxicity, and its semisynthetic derivative, FK317, has been found to be highly potent due to its exceptional DNA cross-linking activity and reduced toxicity [71]. Despite achieving the desirable attributes of selectivity and specificity for successful anti-cancer therapy, MTMs could not overcome the resistance mechanism. Factors such as the insufficiency of activating enzymes like NAD-(P)H oxidoreductase, enhanced drug efflux, and DNA repair processes contribute to the development of resistance in cancerous cells treated with MTMs [71].

Mithramycin and Chromomycin, produced by different species of Streptomycete, are members of the aureolic acid family and are well-recognized for their antitumor potential [72]. Chemically, all members contain glycosylated aromatic polyketides with two oligosaccharide chains of variable length. The isolation of these products was initially prompted by their antibacterial activity against gram-positive bacteria. However, later recognition of their antitumor activity shifted pharmacological interests from antibacterial to antitumor applications. The specificity of mithramycin and chromomycin resembles that of MTMs, as both preferentially interact with DNA minor grooves rich in GC content [73]. The binding, facilitated by Mg^2^⁺, promotes the formation of several hydrogen bonds between aglycone hydroxyl groups and guanine amino protons. This interaction with the DNA double helix impedes RNA synthesis and/or DNA replication [73].

### Topoisomerase inhibitors

Topoisomerase, due to its indispensable role in maintaining DNA integrity during cell proliferation and differentiation, may provide an important strategy to target tumor cells. Drugs such as amsacrine, etoposide, and doxorubicin target topoisomerase type II (type II), while cytotoxic alkaloids and camptothecin (CPT) are recognized as inhibitors of type I [74]. Despite their clinical significance, their use in anticancer therapies is limited due to negative consequences, such as dose-limiting toxicities and drug resistance in the case of type I inhibitors [75]and the development of secondary malignancies when treated with type II inhibitors [76] (Fig. 2). Resistance can also arise from overexpression of pumps like P-glycoprotein, which reduces intracellular drug concentrations, and from modifications in the drug-binding regions of topoisomerase enzymes [77].

CPT, a plant alkaloid produced by *Camptotheca cuminata*, exhibited remarkable anticancer activity in initial clinical trials. However, its limited solubility and adverse side effects have restricted its clinical use. Numerous derivatives of CPT, such as topotecan, have been developed to enhance the drug’s effectiveness [77]. Fluorinated camptothecin derivatives A1 and A2 demonstrated improved antitumor activity against hepatocellular carcinoma, with reduced toxicity compared to topotecan, highlighting their potential as more effective and safer cancer therapeutics [78]. Acridone, analogous to CPT, possesses a planar ring structure that allows it to act as a DNA intercalator and inhibit topoisomerase [79]. Various derivatives of acridone have been synthesized and tested for their antitumoral potential.

Berberine, an isoquinoline quaternary alkaloid extracted from various medicinal plants such as *Hydrastis canadensis* and *Berberis aristata*, is used to treat cancer [80]. Berberine chloride demonstrates therapeutic potential in metabolic-related diseases by upregulating mitochondrial UCP1 expression, promoting thermogenesis, and reducing adipocyte content through ATP depletion and mitochondrial uncoupling. It interacts with DNA, RNA, and other proteins, including telomerase, topoisomerase (type I & II), matrix metalloproteinases (MMP), TP53, and estrogen receptors [81]. Further studies may elucidate its precise mechanisms and applications [82]. Luotonin A is another alkaloid derived from *Peganum nigellastrum*, traditionally used to treat rheumatism, abscesses, and inflammation. Studies on leukemia P-388 cells revealed the antitumorous potential of Luotonin A [83]. This antitumor activity is attributed to its inhibition of type I. Another class, terpyridines (derivatives of pyridine), are also documented as potent inhibitors of type I and II [84].

Quinones, derivatives of aromatic compounds such as benzene and naphthalene, are well known for inducing cytotoxicity. Anthracyclines such as doxorubicin and daunomycin are widely used for cancer treatment. They damage DNA by generating iron-mediated free oxygen radicals [85]. However, life-threatening side effects, such as cardiotoxicity, pose a serious risk for their use in chemotherapy [85]. Multiple anthraquinone derivatives including naphthylquinone and phenanthraquinone have been synthesized and tested for their cytotoxic potential, highlighting their clinical significance [86]. In addition, the ability of quinolone to inhibit DNA gyrase and type IV topoisomerase led researchers to explore its antitumor potential [87]. Several quinolone derivatives have been developed which exhibit moderate antiproliferative activity, while quinophenoxazine analogs demonstrate potent inhibition for type I and II topisomerases [88].

Flavonoids such as fisetin and myricetin also possess anti-topoisomerase activity in cancer cell lines and model organisms. Fisetin and myricetin are dual inhibitors of DNA topoisomerases type I and II [89]. Coumarins, secondary antimetabolites of plants, possess antitumorigenic functions such as blocking the cell cycle, promoting cell death, inhibiting DNA topoisomerase, and modulating estrogen receptors (ER) [90]. Diterpenes isolated from plants, marine sources, and microorganisms also express cytotoxic properties by suppressing topoisomerase activity. Interestingly, fatty acids, including palmitic acid and (5*Z*,9*Z*)-11-phenylundeca-5,9-dienoic acid, also show high anti-topoisomerase activity [86].

Transition metal complexes hold tremendous potential for diagnosis and treatment of cancer due to their high affinity for DNA. Ruthenium (Ru) (II)-polypyridine complexes, Cu (II) complexes, platinum complex, and cobalt (II) complexes are found to be highly effective against cancerous cell lines due to selective inhibition of topoisomerases [86]. Many chemotherapeutic drugs induce senescence in tumor cells, exposing them to an immune surveillance system for elimination. A study on breast cancer patients treated with anthracycline and alkylating agents showed the induction of senescence-associated secretory phenotype (SASP) [91]. In addition to preventing type II, promoting histone eviction, and inducing epigenomic and transcriptomic modifications, doxorubicin induces senescence in cells [92].

Innovations such as CPT derivatives, flavonoids, and transition metal complexes are being explored to minimize adverse effects without sacrificing effectiveness. Emerging compounds such as berberine and quinolone derivatives offer new avenues for targeted therapies with improved safety profiles. Overall, topoisomerase inhibitors remain essential in oncology, with ongoing advancements optimizing their potential for effective cancer treatment.

### Antimitosis drugs

Antimitotic drugs target the mitotic process by inhibiting microtubule assembly or stabilizing microtubules thereby halting cell division and inducing apoptosis or mitotic catastrophe. This strategy is effective because cancer cells often have dysregulated cell cycles and are particularly susceptible to these disruptions. Various mitotic inhibitors are listed in Table 1 and are presumed potential targets for treating cancer are undergoing clinical trials [93]. In addition to approved molecules, many experimental drugs that inhibit and disrupt cell cycle progression are currently being investigated [94–97] (Fig. 2).

Antimitotic drugs that inhibit microtubule assembly, such vinorelbine and gefitinib, have demonstrated notable therapeutic advantages. A randomized study comparing the potency of vinorelbine and gefitinib found no discernible difference between the two in older patients suffering from NCSLC; nevertheless, vinorelbine has been observed to have better tolerability [98]. In a safety and efficacy study, vinorelbine was proven as a good alternative for chemotherapeutic agents in age-enervated patients. Other potent drugs targeting the polymerization of microtubules include vinblastine and vincristine. Vincristine is used in colon and brain cancer, particularly in advanced stages; however, it may induce chemoresistance in patients and has notable side effects, including neurotoxicity and myelosuppression [95, 99].

Docetaxel and paclitaxel stabilize microtubules and are widely used in the treatment of advanced cancers such as prostate, breast, and lung cancer. Docetaxel, along with prednisone, is recommended for patients in an advanced stages of prostate cancer [100]. In most cases, patients develop resistance against docetaxel due to multiple drug resistance mechanisms adapted by cells over time. These mechanisms include enhanced metabolic activity of drug-detoxifying proteins such as glutathione-S-transferase, increased efflux of the drug partially caused by upregulation of ATP-binding cassette (ABC) transporters, changes in microtubule formation and kinetics due to overexpression of βIII-tubulin, and mutations in tumor suppressor genes [101]. Despite the development of resistance, these drugs remain effective in many cases.

Paclitaxel (PTX) is a naturally occurring compound extracted from the Pacific Yew tree and is the first identified microtubule-stabilizing agent with wide-ranging activity in various cancer types [102]. However, the patient response is variable due to genetic infidelity and acquired resistance [103]. Cabazitaxel is a cytotoxic anti-microtubule agent used to treat metastatic prostate cancer patients. A comparative study evaluating the efficacy of prednisone with either cabazitaxel or mitoxantrone in hormone-refractory metastatic prostate cancer patients, previously treated with a docetaxel-containing regimen, showed better OS rate and tumor response rate in patients receiving cabazitaxel [104]. Another randomized study assessed the effectiveness of cabazitaxel and abiraterone or enzalutamide (testosterone-signaling-directed inhibitors) in advanced castration-resistant prostate cancer patients who had previously received docetaxel. Patients treated with cabazitaxel showed survival advantages compared to abiraterone or enzalutamide [105].

Antimitotic drugs can lead to the development of resistance through various mechanisms, including changes in microtubule dynamics, overexpression of drug-efflux pumps, and mutations in tumor suppressor genes. Due to their impact on microtubules, these drugs also exhibit significant side effects, particularly on non-proliferating cells such as neurons [106]. Antimitotic agents are often limited by their toxicity, which can affect both tumor and normal cells. Combining antimitotic drugs with other anticancer agents can enhance their efficacy. For instance, combining antimitotics with targeted therapies or other chemotherapeutic agents has shown promising results in clinical trials [107]. Research is ongoing to develop drugs that target different aspects of mitosis, such as centrosomes and associated proteins, as well as mitosis-associated kinases like cyclin-dependent kinases (CDKs) and Aurora kinases [106]. These approaches aim to improve the selectivity and efficacy of antimitotic therapies while reducing side effects.

### Chemoresistance

Chemotherapy has been a common cancer treatment for decades, but its effectiveness is limited by chemoresistance. Chemoresistance may be acquired, emerging during therapy, or intrinsic, existing prior to treatment as a result of genetic alterations, tumor heterogeneity, and other factors including inadequate drug absorption or metabolism. As dynamic structures, cells continuously integrate mechanical and biochemical information to acclimate to their surroundings. Drugs are taken by cells by two means: passive flow and carrier-mediated translocation. More than 40 ATP-binding cassette (ABC) carriers translocate the drugs across the cell membrane in humans. The expression of these transporters in different tissues influences the cellular response to specific treatments. For example, doxorubicin, used to treat breast cancer, bladder cancer, lymphoma, and acute lymphoblastic leukemia (ALL), is less effective in cells expressing p-glycoprotein (p-gp), as p-gp pumps the drug out, protecting cells from ER stress [108]. Therefore, variations in p-gp expression can impact chemotherapy success. Similarly, the expression of other transporters affects the uptake and retention of cytotoxic drugs in tumor cells.

Limited metabolic activation of prodrugs and/or increased inactivation of drugs also leads to the development of drug resistance. Anti-tumorous medicines are metabolized by enzymes in the intestine, liver and tumor. Human cytochrome P450s (CYPs) oxidize drugs like dacarbazine, procarbazine cyclophosphamide, ifosfamide and tamoxifen to active cytotoxic form. Cytochromes like CYP3As detoxify chemotherapeutic drugs such as tamoxifen, imatinib, sorafenib and gefitinib, etc. Tumors with elevated expression of CYP3As decrease drug efficacy [109]. Glutathione S-transferase (GSTs), UDP glucuronosyltransferases (UGT), methyltransferases, sulfotransferases and N-acetyltransferase metabolize the antineoplastic drugs [110]. Hence, the activity of these enzymes, influenced by genetic and epigenetic factors, regulates the cellular response to the therapy.

Furthermore, drug resistance is caused by dysregulation of molecular pathways involved in metabolism, cell proliferation, apoptosis, and autophagy. Epithelial to mesenchymal transition (EMT)—a crucial step in metastasis-related transcription factors (EMT-TFs) such as Snail, Twist, Slug, Forkhead box C2 (FOXC2) and Zinc finger E-box binding homeobox 1 (ZEB1) induce drug resistance [111]. BCL-2 family has pro-apoptotic (Bax, Bak, Bid, Bad, Bim, Noxa and Puma) and anti-apoptotic (BCL-2, BCL-W, BCL-XL, MCL-1, and BFL-1/A1) members. Cells’ decision to live and die depends on the balance of pro-apoptotic and anti-apoptotic proteins. Anti-apoptotic pathways are generally upregulated in cancers to help chemoresistance and evasion from apoptosis [112]. Chemotherapeutic drugs kill cancerous cells by damaging its DNA while cells have repair methods to reverse the damage induced by chemotherapeutic medicines. Upregulation of DNA damage repair pathways also helps cancerous cells to survive stress. Studies demonstrate that doxorubicin resistance can be overcome by inhibiting DNA repair kinases [113]. Expression of MRE11—a DNA damage response protein—determines the outcome of chemotherapy with the probable increase in chemoresistance [114].

In a nutshell, tumor heterogeneity is the primary factor giving rise to chemoresistance. Early-stage tumors are small and less heterogeneous; hence can be managed by different therapies. With the increase in size and prolonged exposure to a specific chemotherapeutic drug, chemoresistance arises, requiring reconsidering administered therapy. In addition, a profound understanding of genetic mutation present in tumor tissue, mechanism of action of drugs, development of resistance against drugs and ways to overcome drug resistance would enhance the likelihood of therapeutic achievements.

## Hormone therapy

Hormonal therapy is a crucial approach for hormone-dependent tumors, including breast, prostate, and ovarian cancers (Table 2).

**Table 2:** List of hormonal therapy agents for cancer treatment

Process	Mechanism of action	Agents
*Breast and ovarian cancers*		
Ovarian extirpation	Surgically removes ovaries to eliminate estrogen source	
GnRH antagonism	Blocks GnRH receptors to prevent estrogen production	
Estrogen receptor blocking	Blocks estrogen receptors, preventing estrogen from binding and activating them	Tamoxifen, toremifene, fulvestrant
Aromatase inhibitors	Inhibits the conversion of other hormones (like androgens) into estrogen	Letrozole, anastrozole, exemestane, goserelin
*Prostate cancer*		
Surgical castration	Surgically remove testicles to stop production of testosterone	
Androgen deprivation therapy	Reduces or stops testosterone production	
LHRH antagonism	Inhibits production of testosterone by blocking luteinizing hormone (LH) release	Bicalutamide
GnRH antagonism	Binds to GnRH receptors in pituitary glands and prevent its interaction with GnRH	Degarelix, relugolix, leuprolide acetate

*GnRH* gonadotropin-releasing hormone, *LHRH* luteinizing hormone-releasing hormone, *LH* luteinizing hormone

### Breast cancer

In breast cancer, early diagnosis is vital for better outcomes. Since 60–70% of breast cancers express estrogen receptors, therapies that control the estrogen levels have proven effective. For premenopausal females with early-stage tumors, ovarian extirpation is an option but comes with high morbidity. A less invasive alternative is luteinizing hormone-releasing hormone therapy (LHRH), which inhibits estrogen production in the ovaries. Goserelin acetate, a decapeptide analog of LHRH, is one of the most prescribed agonists in premenopausal patients with primary and advanced stages of cancer [115]. Alongside selective estrogen receptor modulators (SERM), such as tamoxifen has proven effective. SERM occupies the ligand binding domain (LBD) of estrogen receptor (ER), influencing its interaction with co-activators and inhibitors. However, tamoxifen treatment often leads to thromboembolism [116]. Hence, alternatives like toremifene have been developed [117]. Notably, a comparative study on postmenopausal patients with node-positive breast cancer found no significant difference in disease-free survival (DFS), OS, or side-effect profiles between toremifene and tamoxifen [118]. In tamoxifen-resistant cases, selective estrogen receptor down-regulators (SERD) have shown effectiveness by inducing degradation and disrupting the ER signaling cascade [119].

In postmenopausal females, androgens secreted by adrenal glands are converted into estrogen by aromatase, which is mainly produced by fatty tissues [120]. Aromatase inhibitors (AIs) reduce estrogen levels by blocking the activity of aromatase (an enzyme responsible for converting androgens to estrogen). AIs and ovarian suppression agents offer alternatives with fewer side effects compared to tamoxifen. AI drugs, such as letrozole, anastrozole, and exemestane, are designed with high specificity for aromatase and inhibit aromatization by over 99%. However, their response rates range from 35 to 70% in neoadjuvant studies, and decreased efficacy may be observed in advanced stages of the disease [121].

Additionally, the development of resistance after prolonged exposure to treatment is a key obstacle in cancer therapy [122]. Resistance to AIs can be overcome by targeting type I growth factor receptors such as EGFR, HER-2, and mTOR inhibitors [123]. A study evaluating the combination of letrozole (aromatase inhibitor) with everolimus (mTOR inhibitor) compared to letrozole alone showed reduction in tumor size and increased sensitivity in the combination treatment. This suggests that the AKT pathway may play a role in resistance to letrozole [124].

Genetic polymorphism can also impact the functionality of AIs. A study highlighted the role of SNP single nucleotide polymorphism (rs6493497 and rs7176005) in regulating the activity of aromatase (CYP19) following AI treatment. In hormone receptor-positive metastatic breast cancer patients, SNP (rs4646) of the aromatase CYP19 gene was significantly associated with treatment efficacy [125]. A comparative study examining the role of different proteins in patients treated with letrozole alone or in combination with chemotherapy observed the resistive role of HIF1-α (hypoxia-inducible factor 1-α) and P44/42 mitogen-activated protein kinase (MAPK) [126]. It is worth noting that AIs induced suppression of estrogen is endogenous; therefore, the production of other steroids or their interaction with ER and exogenous estrogen entering the system due to industrial pollution, synthetic or phytoestrogen, remains uninfluenced.

Among other treatment options, targeting the HER family of growth factor receptors through antigrowth factor receptor antibodies (e.g., trastuzumab) or small molecule tyrosine-kinase inhibitors (e.g., gefitinib and lapatinib) is effective. In a randomized trial, ER-positive advanced breast cancer patients treated with gefitinib plus anastrozole experienced longer progression-free survival (PFS) compared to those receiving only anastrozole [124]. Similar results were obtained when patients with known ER-positive/HER-positive status were treated with lapatinib added to letrozole, signifying the role of HER2 in limiting the effectiveness of Ais [127].

### Ovarian cancer

Hormone receptor expression, particularly estrogen receptors (ER) and progesterone receptors (PR), plays a crucial role in the treatment and outcomes of ovarian cancer. However, the expression of these receptors varies significantly among different subtypes of ovarian cancer. For instance, serous carcinomas, the most common subtype, often exhibit higher ER and PR expression levels [128]. In contrast, mucinous and clear cell carcinomas may have lower levels of these receptors [129]. These differences in receptor expression between subtypes can significantly influence the responsiveness to hormone therapy.

The variability and often low expression of hormone receptors in ovarian cancer pose significant challenges for hormone therapy. The lack of hormone receptor expression in a substantial proportion of ovarian cancer cases limits the efficacy of hormone therapies. However, numerous studies assessing tamoxifen’s effectiveness for epithelial ovarian cancer found that the drug stabilized the disease in 30–40% of cases, although response rate was poor i.e., 10–15% [130, 131]. Similarly, clinical trials of anastrozole (AI) conducted on ER and PR-positive ovarian cancer patients—pre-treated with chemotherapy and having limited disease, also yielded disappointing outcomes [132]. In contrast, tamoxifen treatment for Chinese ovarian cancer patients with advanced chemo-resistant disease achieved PFS with limited side effects [133]. A comparative study evaluating the clinical significance of tamoxifen and letrozole for advanced epithelial ovarian cancer concluded an equivalent overall response rate, disease stability rate and clinical benefit rate. Letrozole, however, was found to have a longer response duration than tamoxifen (26 vs. 11.5 months) [134]. Identifying patients who will benefit from hormone therapy is challenging due to the lack of reliable biomarkers. Determining hormone receptor status is essential for guiding treatment and minimizing unnecessary side effects.

Even in patients with initially responsive tumors, acquired resistance to hormone therapies is a common issue. Resistance can develop through various mechanisms such as alterations in hormone receptor signaling pathways, changes in co-regulator proteins, or compensatory upregulation of alternative pathways. For example, activation of the PI3K/AKT pathway may contribute to resistance against hormone therapy. The development of resistance can lead to disease progression, necessitating alternative therapeutic strategies. This highlights the need for continuous monitoring and adaptation of treatment plans [135].

Combining hormone therapy with chemotherapy or targeted therapies may improve outcomes in hormone-sensitive ovarian cancer. For instance, using LHRH agonists like goserelin acetate in conjunction with chemotherapy can be beneficial in premenopausal patients. Several clinical trials are investigating combination regimens to identify optimal strategies that overcome resistance and improve patient outcomes.

Further research is needed to fully understand the role of hormone therapy in ovarian cancer. Studies exploring the molecular mechanisms behind hormone receptor signaling and resistance are crucial. This includes investigating co-regulator proteins and alternative pathways that may contribute to resistance. Developing novel agents targeting specific pathways involved in hormone receptor signaling, or those that can effectively target tumors lacking traditional hormone receptors, holds promise. For example, targeting the PI3K/AKT pathway or developing new SERMs and aromatase inhibitors with enhanced efficacy and fewer side effects could be beneficial.

### Prostate cancer

Prostate cancer is the second most prevalent cancer in males and the fifth leading cause of death among men globally. Despite the high incidence, limited therapeutic choices are available [136]. Since most prostate cancers require androgens for growth, androgen deprivation therapy through either orchiectomy (surgical castration) or estrogen (chemical castration) is the standard first-line treatment for advanced prostate cancer [137].

Estrogen (diethylstilbestrol) was used for medical castration as an alternative to orchiectomy to suppress testosterone levels. However, cardiovascular side effects decreased its consumption [138]. Researchers began searching for antiandrogenic compounds competing with androgens for binding to receptors in the nucleus and inducing apoptosis. Multiple antiandrogenic steroids were tested; however, a meta-analysis of 2717 patients suggested a decrease in OS rate with the use of non-steroidal antiandrogen monotherapy compared to surgical castration [139]. A randomized trial of bicalutamide (non-steroidal antiandrogen) in 1435 patients revealed its low efficiency in patients with M1 disease and no significant difference in OS with locally advanced M0 disease compared to surgical castration [140].

LHRH agonists have become the standard for modern androgen deprivation therapy (ADT) as a practical and reversible alternative to orchiectomy. A study analyzed the outcome of 10 trials from 1908 patients concluded the consistency in OS rate in response to GnRH (Gonadotropin hormone-releasing hormone) agonist treatment with orchiectomy or diethylstilbestrol in advanced disease [139]. Furthermore, GnRH agonist proved effective solution for early-stage prostate cancer. When given to patients for 3 years after radiotherapeutic treatment, GnRH agonists improved DFS and OS for up to 10 years in high-risk metastatic patients [141]. However, GnRH agonist therapy can lead to severe health risks, including bone pain, spinal cord compression, ureteral obstruction, and possibly death due to an sudden increase in testosterone levels following GnRH receptors stimulation [142].

Researchers identified GnRH antagonists that can bind to and block GnRH receptors. Abarelix was the first FDA-approved GnRH antagonist that was found to be effective in castration without causing testosterone surge [143]. However, continued use of abarelix diminished its testosterone-suppressive effect in two clinical trials [142]. In the European phase III trial, abarelix treatment was more effective at avoiding castration than other antiandrogenic compounds [143]. Despite this, the risk of immediate-onset histamine-mediated hypersensitivity reactions limited its clinical utility, and abarelix was withdrawn from the US market in 2005 [144].

Degarelix, an approved ADT, suppresses luteinizing hormone (LH) and follicle-stimulating hormone (FSH) by antagonizing GnRH. In a year-wide randomized study that enrolled 610 patients, effective repression of testosterone without an initial surge was observed. The treatment also resulted in an immediate decrease in testosterone and prostate-specific antigen (PSA) levels, improved overall survival, and a reduction in hormone-refractory disease [145]. However, the patients treated with degarelix experienced injection site reactions, chills, urinary tract infection, and musculoskeletal complications [146]. The need for periodic administration and high occurrence (~ 40%) of injection site reaction have limited the clinical applications of degarelix [145].

Relugolix is an alternative GnRH antagonist developed for oral administration with high selectivity and a longer half-life of 25 h to avoid complications of degarelix [146]. Various phase I and II studies asseverate the rapid activity of relugolix in inhibiting the release of FSH and LH from the pituitary and lowering testosterone levels [147]. In a randomized phase III study, leuprolide acetate (22.5 mg by injection after 90 days), a GnRH analog with a half-life of 3 h reduced testosterone levels as compared to relugolix (120 mg one time a day after an initial oral dose of 360 mg). Testosterone surge was noticed in the leuprolide group, while the group with relugolix treatment did not show a rise in testosterone before reaching castrate level. Additionally, relugolix treatment eliminated the need for antiandrogens to mitigate undesirable effects [148].

**Fig. 3:**
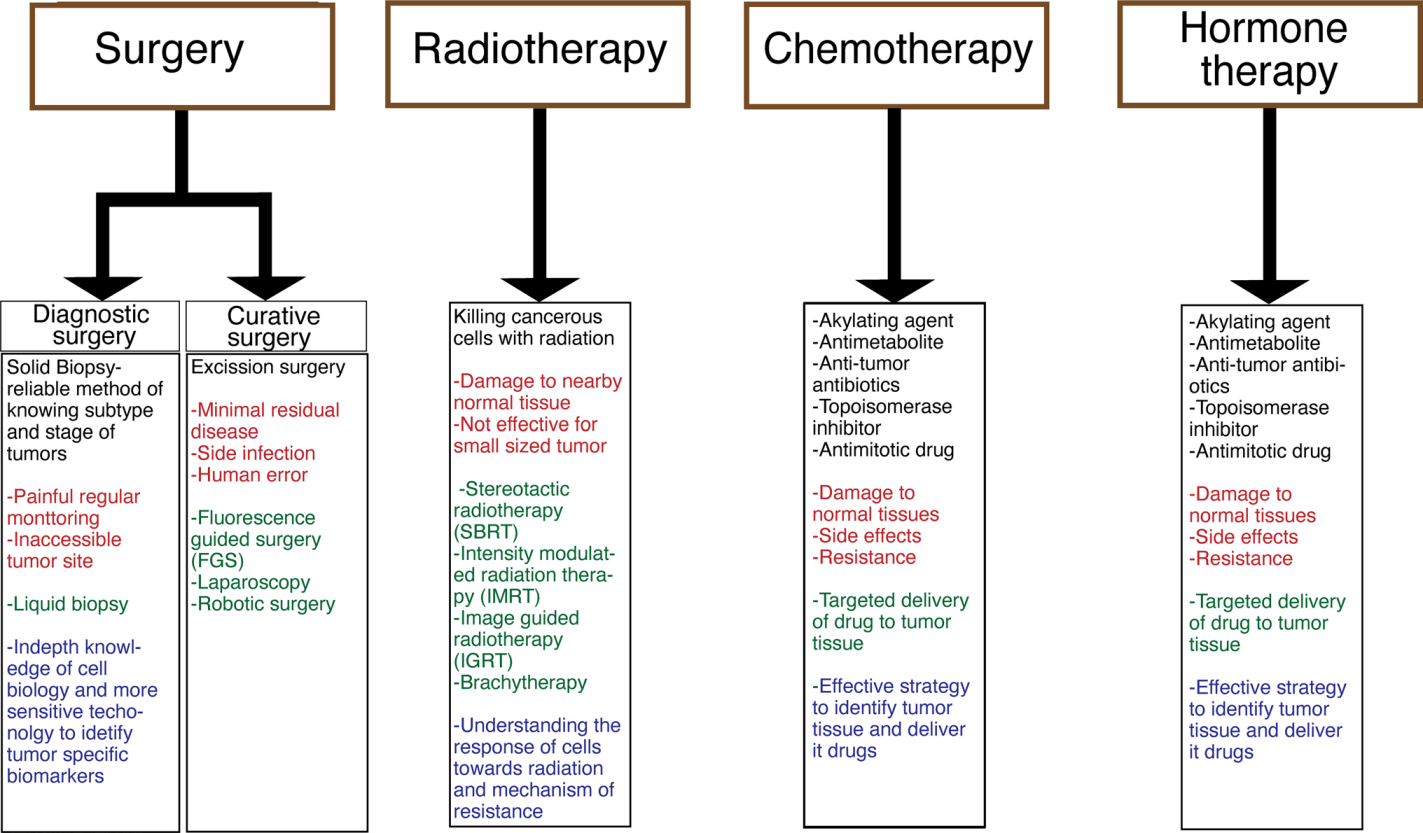
Evolution of traditional therapies: past, present, and future directions, illustrated through different font colors. The black font categorizes the techniques for each anti-cancer treatment. The red font highlights the challenges associated with these therapies. Green font represents the innovations made to overcome these challenges, while the blue font emphasizes the future directions aimed at enhancing the efficiency of specific therapies

In summary, Fig. 3 effectively encapsulates the evolution of traditional therapies in the context of anti-cancer treatments, highlighting the intricate interplay between established techniques, ongoing challenges, and innovative solutions. As we move forward, it is essential to continue fostering innovation and collaboration in the field, ensuring that the progress made today paves the way for more effective and accessible cancer therapies in the future.

## Conclusion

With advancements in science and technology, traditional cancer treatments have significantly improved, offering greater efficiency and precision. However, despite these advances, cancer therapy continues to face several key challenges across various treatment modalities. Surgery remains effective for localized tumors, but its success can be limited by factors such as tumor size, location, and metastasis. Moreover, surgery carries inherent risks and may not be suitable for all patients, particularly those with advanced disease. Chemotherapy and hormone therapy are frequently hindered by resistance mechanisms—chemoresistance and hormone resistance—resulting from genetic mutations, alterations in signaling pathways, or tumor heterogeneity. These resistances reduce the long-term effectiveness of treatments, underscoring the need for novel therapies or combination strategies to overcome them. Many cancer therapies, including antimitotic drugs, chemotherapy, and hormone therapies, are associated with significant side effects due to their impact on both cancerous and healthy cells. For example, antimitotic drugs can cause neurotoxicity, chemotherapy often leads to gastrointestinal, hematologic, and systemic toxicities, and hormone therapies may induce thromboembolism or cardiovascular issues, limiting their long-term use and patient compliance.

The effectiveness of these treatments is often variable among patients, influenced by genetic polymorphisms, tumor heterogeneity, and the evolving nature of cancer. This highlights the need for personalized medicine and better biomarkers to guide treatment decisions. Traditional therapies, such as ADT for prostate cancer and AIs or SERMs for breast cancer, often have limited response rates and are prone to the development of resistance. Additionally, newer therapies like GnRH antagonists and oral therapies offer promise but still face challenges, including side effects and limited long-term efficacy. There is a clear need for new drugs targeting previously unexplored aspects of cancer biology, such as mitotic kinesins, Aurora kinases, and mTOR inhibitors. However, combination therapies that integrate different treatment modalities may improve efficacy by synergizing their strengths and minimizing resistance and side effects. Still, the complexity of drug interactions and the risk of cumulative toxicity complicate the development and clinical application of such therapies.

## Data Availability

No datasets were generated or analysed during the current study.
